# The Rural Population

**Published:** 1918-10-26

**Authors:** 


### THE RURAL POPULATION.

The ravages of the war and the falling birth-rate call for increased effort towards conserving and increasing the rural population of England. In the rural districts there is a better standard of health and physical well-being, while the infant mortality is lower; and that notwithstanding the fact that in many rural districts the internal home conditions, more especially the bedroom accommodation and cleansing facilities, compare very unfavourably with those in urban districts. Apart from such important questions as food production and rural industries it cannot be too definitely realised that the national health standard, which has been seriously impaired in consequence of the drain on the virile manhood of the nation, will most rapidly and most efficiently be raised by increasing the rural population and improving the conditions of rural home life. For obvious reasons many people prefer town life to country life, so that it is necessary to provide inducements and incentives to live in the country. Such inducements include good wages, good housing accommodation, educational facilities, increased transport and travelling facilities and means for amusement and recreation. The rural population is the chief source to which we must look for a higher and increasing birth-rate, and in order to replenish the wastage in human life which has resulted from the war every effort must be made to revive and increase the life and activity of rural England.

